# A Rare Case of Tuberculosis Revealed by Acute Appendicitis: A Case Report

**DOI:** 10.7759/cureus.51733

**Published:** 2024-01-06

**Authors:** Fouad Haddad, Malak Afifi, Fatima Zahra El Rhaoussi, Mohamed Tahiri, Wafaa Hliwa, Ahmed Bellabah, Sanaa Hachimi, Nisrine Bennani Guebessi, Badre Wafaa

**Affiliations:** 1 Gastroenterology and Hepatology, Ibn Rochd University Hospital Center, Casablanca, MAR; 2 Medicine and Pharmacy, Hassan II University, Casablanca, MAR; 3 Anatomical Pathology, Ibn Rochd University Hospital Center, Casablanca, MAR

**Keywords:** acute appendicitis, morocco, epithelioid granuloma, extrapulmonary tuberculosis (eptb), appendicular tuberculosis

## Abstract

According to the World Health Organization (WHO), tuberculosis (TB) is the 13th cause of death worldwide and the second infectious killer after HIV. It is an endemic disease in Morocco. Isolated appendicular TB is an uncommon form of extrapulmonary TB. We report a case of a 26-year-old woman admitted for acute abdominal pain in the right iliac fossa with fever, vomiting, and diarrhea. Physical examination and abdominal ultrasound confirmed appendicitis. Surgery was performed and revealed on histopathological examination of the resected appendix the diagnosis of tubercular appendicitis. The patient was initiated on the conventional antitubercular regimen for six months and would be followed up appropriately. This case report highlights the importance of histopathological examination of appendicectomy specimens in order to diagnose rare diseases such as primary TB of the appendix.

## Introduction

Tuberculosis (TB) is an endemic disease in Morocco. In 2021, 29,327 cases were identified which corresponds to an incidence rate of 80 new cases per 100,000 inhabitants, all forms combined. Mycobacterium TB is the causative agent of TB. Pulmonary TB is the most common site involved with a number of 5.3 million people worldwide in 2021 [1]. Gastrointestinal TB represents 3% of extrapulmonary TB and concerns primarily the ileocecal region [2]. Primary TB of the appendix is a rare entity with a prevalence of 0.1% to 3% in all appendicectomies performed [3]. The aim of this study is to report a case of appendicular TB revealed by appendicular peritonitis in the emergency department of the Ibn Rochd University Hospital Center of Casablanca (Morocco) in 2023.

## Case presentation

A 26-year-old young Moroccan female was admitted to the emergency department of Ibn Rochd University Hospital Center of Casablanca for acute abdominal pain in the right iliac fossa for two days. She also reported vomiting and diarrhea (three to four times a day) along with fever, night sweats, and a weight loss of five kilograms in a month. Her medical and familial history was not significant. She had no past history of TB. She has been vaccinated against TB as per the national immunization program.

On examination, she was conscious, febrile (38.5°C) and tachycardic (100 /min). Her blood pressure was 120/70 mmHg. The abdominal examination noted rebound tenderness in the right iliac fossa over the McBurney point without any palpable mass. It also reported flank dullness. Washboard abdomen was not found. The rectal examination did not reveal nodules, masses, or tenderness. Examination of her respiratory and cardiovascular systems was normal.

Blood tests revealed microcytic and hypochromic anemia with 9.6 g/dL of hemoglobin (normal range 12-16 g/dL), and hyperleukocytosis with a count of 15.7 x 10^9^ (normal range 4.5-11 x 10^9^) with neutrophilia (13.6 x 10^9^) and no lymphopenia. C-reactive protein (CRP) was elevated to 115.4 mg/L (normal range < 1 mg/L). The rest of the blood investigations were normal. The MANTRELS score, also recognized as the ALVARADO score, was 8. A score of 5 or 6 is indicative of acute appendicitis, while a score of 7 or 8 suggests probable appendicitis and a score of 9 or 10 indicates highly probable appendicitis. Abdominal ultrasonography was performed and showed an enlarged and inflamed appendix of 8.6 mm long complicated by peritonitis. There were multiple lymph nodes in the central mesentery artery and abundant ascites.

**Figure 1:**
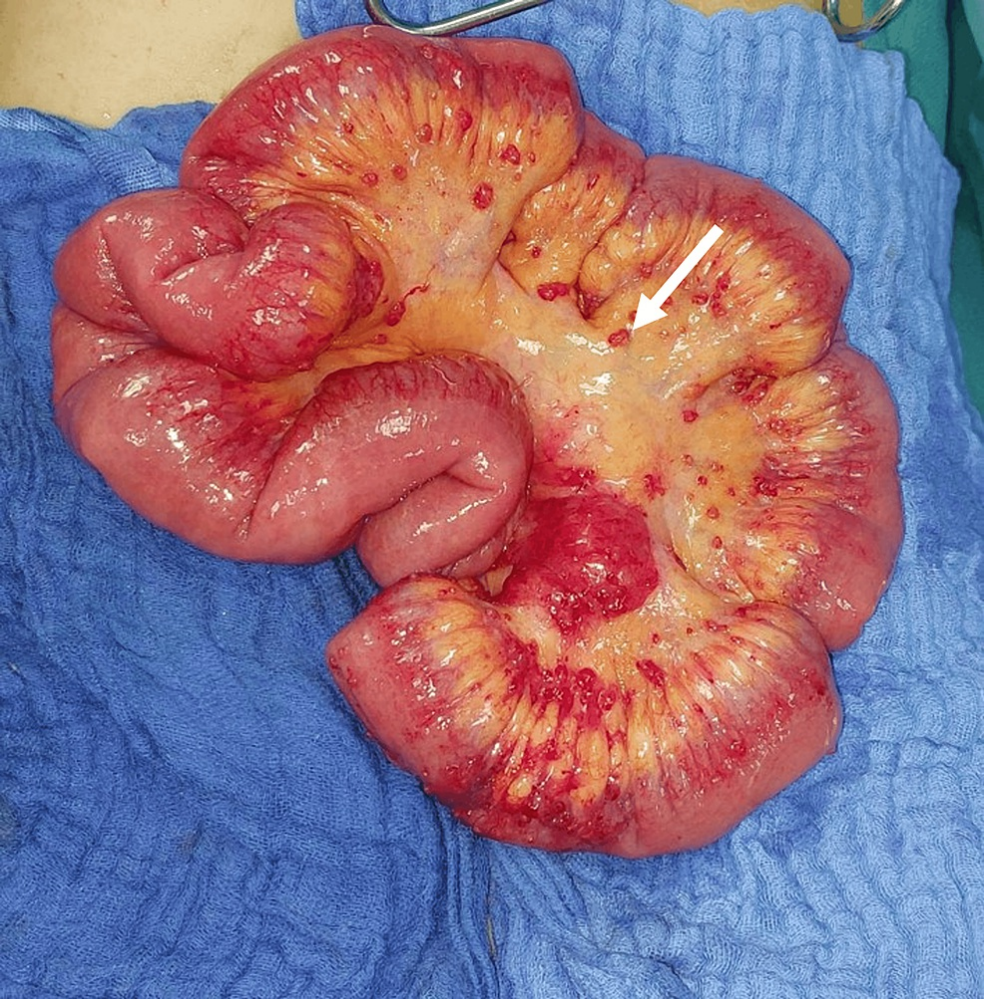
Image of mesenteric granulations suggestive of tuberculosis

**Figure 2:**
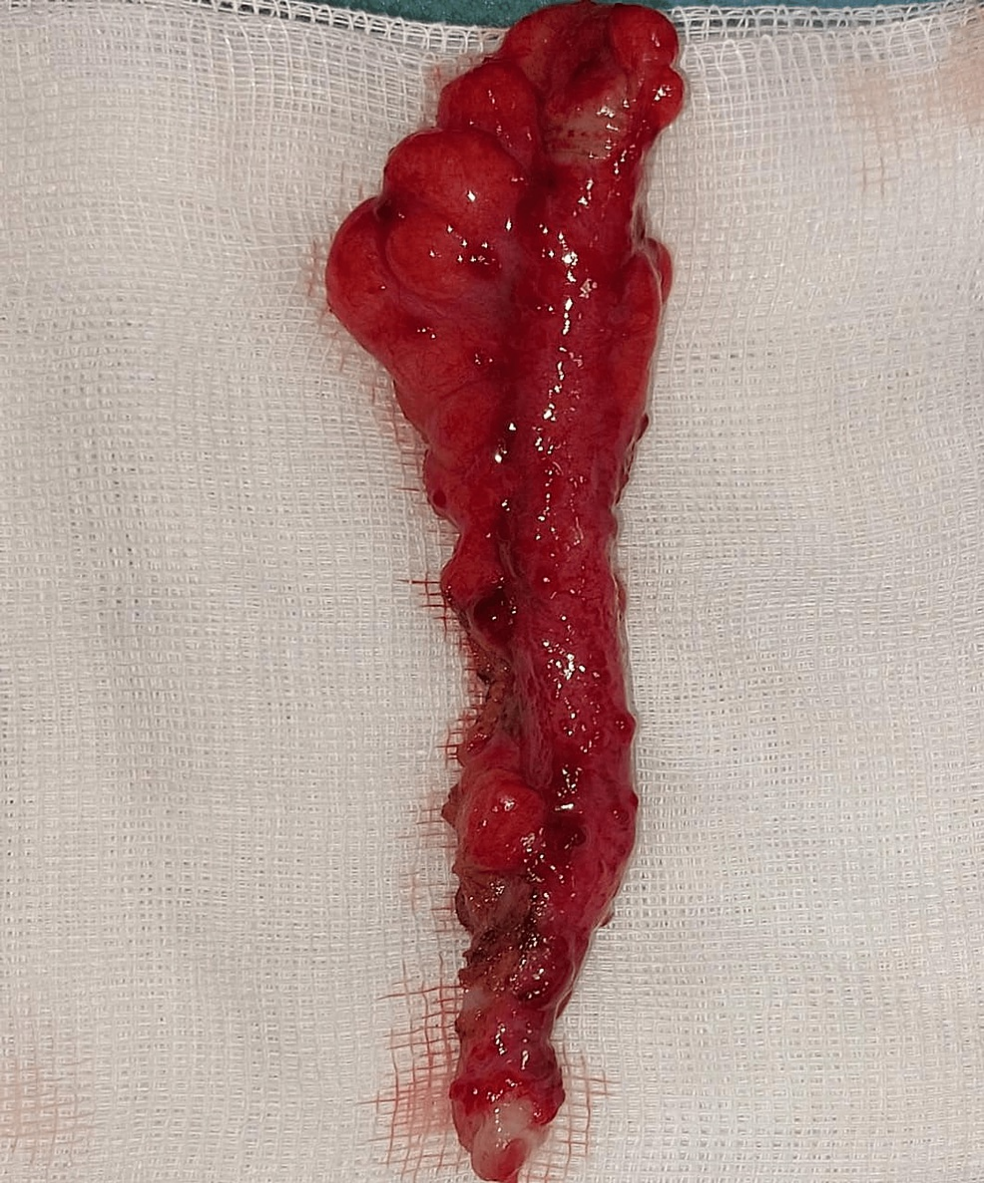
Post-operative picture of the inflamed appendix

The patient was subjected to a laparoscopic appendicectomy for acute appendicular peritonitis. On peritoneal cavity exploration, multiple inflamed nodules were observed in the ileum, the caecum, the large omentum and mesentery. Ascites was explored and evacuated. The appendix was in laterocaecal position. Appendicectomy and peritoneal cleansing were performed, and the specimen was sent for histopathological examination (Figures 1, 2).

Macroscopically, the swollen inflamed appendix measured 8.5cm in lenght and 1cm of diameter (normal diameter < 6mm). Cut surface showed white pseudomembranes. The lumen was obliterated by an appendicolith.

**Figure 3:**
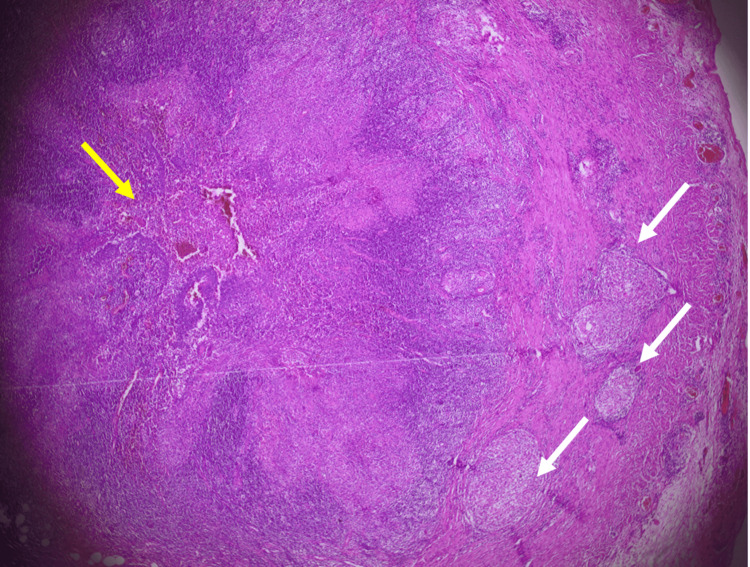
Histological slide of an appendix wall containing epithelioid cell granulomas with foci of caseous necrosis White arrows: Epithelioid cell granulomas, Yellow arrow: Caseous necrosis

**Figure 4:**
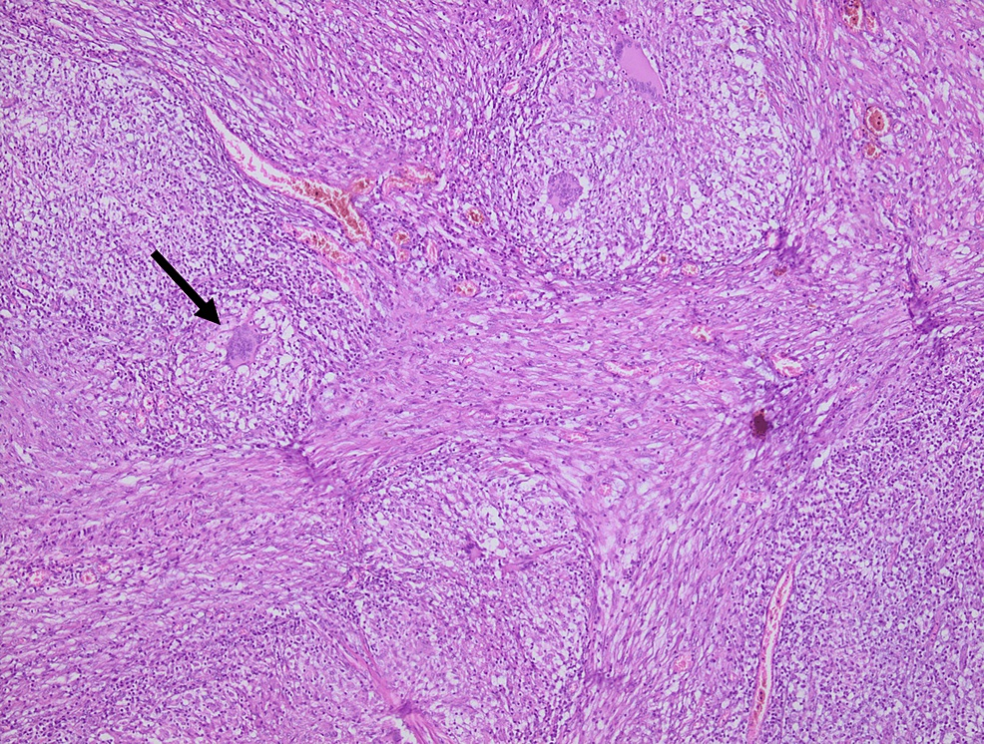
Histopathologic aspects of tuberculosis: multiple epithelioid granulomas with giant cells (arrow)

On microscopic examination, appendix sections revealed along in the thickened wall, multiple gigantocellular epithelioid granulomas and central caseous necrosis. Ziehl Neelsen stain for acid fast bacilli was noncontributory. Based on the location, the diagnosis of tubercular appendicitis was confirmed (Figures 3, 4).

Further investigations were performed. Sputum examination for acid-fast bacillus was negative. Intradermal tuberculin reaction was positive (16mm) (normal range < 15mm for a vaccinated person). Ascites paracentesis revealed a turbid exudative fluid with a high adenosine desaminase activity (86.5 U/L). Chest x-ray showed no abnormality. HIV status was negative.

Antitubercular treatment was started for nine months with rifampicin (10 mg/kg/day), isonizid (5 mg/kg/day), pyrazinamid (30 mg/kg/day) and ethambutol (20 mg/kg/day) for two months followed by seven months of isonizid and rifampicin (2HRZE/7HR), on a daily basis.

During the follow-up visits, the patient had a favorable postoperative recovery. The surgical scar was clean, and healed successfully. The patient tolerated the antitubercular treatment with no side effects. The abdominal symptoms were resolved within two months and ascites disappeared. A full recovery has been marked to date by the end of the treatment.

## Discussion

The incidence of appendicular TB varies from 0.1% to 3% of the appendicectomies performed [3]. Appendicular TB is a rare and less-known entity. It has been described for the first time in the literature by Corbin in 1873. In Morocco, gastrointestinal TB is usual and the commonest site is ileocecal [4]. However, appendix infiltration by the bacilli is rare and may be due to the minimal contact of appendicular mucosa with intestinal contents [5,6].

The pathogenesis of appendicular TB remains uncertain [7]. Tubercular bacilli might infiltrate the digestive tract through various routes, including the ingestion of infected sputum resulting from pulmonary TB or contaminated food (intraluminal, hematogenous, local extension) [8]. Alternatively, hematogenous spread may occur from pre-existing infective sites or through direct extension from the ileocecal region or genital TB [9]. Three clinical types of tubercular appendicitis have been documented in the literature [10]. The initial type is a chronic form characterized by abdominal pain, diarrhea, and occasional vomiting. This form is often mistaken for ileocecal TB [11]. The second type is an acute form with symptoms resembling surgical abdomen appendicitis, as observed in our patient [12]. The third type is latent and asymptomatic, typically diagnosed through histopathological examination [13,14]. Mathew et al. have recently described a unique form that combines appendicular TB with stump appendicitis - a rare and delayed complication of appendicectomy, where the residual stump becomes inflamed [15].

The pre-operative diagnosis of appendicular TB is challenging as there are no pathognomonic clinical, biological, or radiological features to suggest it [16,17]. Consequently, confirmation of the diagnosis can only be achieved through histopathological examination [13]. This case report is consistent with existing literature findings.

Macroscopically, the appendix can be normal, ulcerative, or hypertrophied [18]. Histopathological examination reveals a tuberculous granuloma with central caseous necrosis surrounded by multiple Langhans-type giant cells located in the appendix, which is pathognomonic of TB (Figures 3, 4). Hence, granulomatous appendicitis can be linked to various diseases, including infectious conditions such as Yersinia Enterolitica and parasitic infections. Additionally, non-infectious causes, like Crohn's disease, sarcoidosis, diverticulitis, and the presence of foreign bodies, may also be associated [2,19,20].

Although appendicitis is commonly managed by all physicians, the existence of ascites and mesenteric nodes should lead the treating clinician to consider TB as the principal concern. The treatment of appendicular TB is both surgical and medical [20]. The patient must be given multiple anti-TB drugs according to the World Health Organization with rifampicin, isoniazid, pyrazinamid, and ethambutol for two months followed by seven months of isoniazid and rifampicin (2HRZE/7HR) [4,5]. Appendicectomy is typically performed to avoid inflammation and complications. However, in our case and several literature reviews, the diagnosis was established only after histopathological examination.

## Conclusions

Appendicular TB is a rare entity even in endemic countries. Our clinical case study is consistent with the information available in the literature. It may be associated with TB symptoms, but no specific clinical features exist. It may mimic acute appendicitis and be diagnosed only after histopathological examination. Therefore, surgeons and pathologists should be aware of the possibility of tubercular infection of the appendix in order to start the antitubercular treatment as soon as possible.
